# *Salmonella* Biofilm Formation on *Aspergillus niger* Involves Cellulose – Chitin Interactions

**DOI:** 10.1371/journal.pone.0025553

**Published:** 2011-10-07

**Authors:** Maria T. Brandl, Michelle Q. Carter, Craig T. Parker, Matthew R. Chapman, Steven Huynh, Yaguang Zhou

**Affiliations:** 1 Produce Safety and Microbiology Research Unit, Agricultural Research Service, U.S. Department of Agriculture, Albany, California, United States of America; 2 Department of Molecular, Cellular, and Developmental Biology, University of Michigan, Ann Arbor, Michigan, United States of America; University of Birmingham, United Kingdom

## Abstract

*Salmonella* cycles between host and nonhost environments, where it can become an active member of complex microbial communities. The role of fungi in the environmental adaptation of enteric pathogens remains relatively unexplored. We have discovered that *S. enterica* Typhimurium rapidly attaches to and forms biofilms on the hyphae of the common fungus, *Aspergillus niger*. Several *Salmonella enterica* serovars displayed a similar interaction, whereas other bacterial species were unable to bind to the fungus. Bacterial attachment to chitin, a major constituent of fungal cell walls, mirrored this specificity. Pre-incubation of *S.* Typhimurium with N-acetylglucosamine, the monomeric component of chitin, reduced binding to chitin beads by as much as 727-fold and inhibited attachment to *A. niger* hyphae considerably. A cellulose-deficient mutant of *S.* Typhimurium failed to attach to chitin beads and to the fungus. Complementation of this mutant with the cellulose operon restored binding to chitin beads to 79% of that of the parental strain and allowed for attachment and biofilm formation on *A. niger*, indicating that cellulose is involved in bacterial attachment to the fungus via the chitin component of its cell wall. In contrast to cellulose, *S*. Typhimurium curli fimbriae were not required for attachment and biofilm development on the hyphae but were critical for its stability. Our results suggest that cellulose–chitin interactions are required for the production of mixed *Salmonella-A. niger* biofilms, and support the hypothesis that encounters with chitinaceous alternate hosts may contribute to the ecological success of human pathogens.

## Introduction

Enteropathogenic bacteria persist in the environment where they may interact closely with other members of microbial communities. The contribution of fungi to the ecological success of foodborne pathogens remains unexplored. However, interactions between fungi and human pathogens have been described previously. For example, *Pseudomonas aeruginosa* has been shown to colonize filamentous cells of *Candida albicans*, which leads to biofilm formation followed by filament death [1]. Type IV pili and other virulence factors in *P. aeruginosa* are implicated in this antagonistic effect [1]. Additionally, several bacterial species can inhibit or kill phytopathogenic fungi, thereby making them potentially useful agents for the control of plant disease [2]. Bacterial antagonism toward fungi is frequently mediated by bacterial production of secondary metabolites, although more advanced types of interactions involving the type III secretion system [2] or the production of lytic enzymes that degrade the fungal cell wall [3], [4] have been reported. Other bacterial species are beneficial to their fungal host, such as mycorrhiza-helper bacteria, which promote the symbiotic activity between the mycorrhizal fungus and the plant [5], [6], and *Klebsiella aerogenes*, which provides substrates for melanization in *Cryptococcus neoformans* [7]. In addition, endophytic bacteria have been observed in endo- and ectomycorrhizae [8], [9]. Hence, a great diversity of interactions between bacteria and fungi exists in the environment. Besides potentially providing bacterial colonists with substrates or favorable conditions, filamentous fungi may also facilitate their movement to microsites that would otherwise be unreachable due to a lack of free water. It is likely that over evolutionary time, such intertrophic encounters have shaped the creation of new niches that contribute to the persistence of human pathogens in nonhost habitats.

*Salmonella enterica* colonizes a wide variety of organisms and can survive for prolonged periods of time in soil, sediment, and water, which may facilitate its cycling between host and nonhost environments [10]. A strain of *S. enterica* Enteritidis that caused an outbreak linked to raw almonds persisted for at least five years on the implicated orchard floor [11]. The biotic factors that contribute to the survival of *S. enterica* in the environment remain largely unknown. In particular, the role of fungi in the ecology of *S. enterica* has not been investigated. We provide here, the first report of the intimate association of *S. enterica* with *Aspergillus niger*, a successful colonizer of plants and soil, and a common member of microbial consortia. Unlike other bacterial species, such as *Escherichia coli*, *Pantoea agglomerans*, and *Pseudomonas chlororaphis*, all tested serovars of *S. enterica* attached rapidly to, and formed large and complex biofilms on *A. niger* hyphae. Our results suggest that this association depends on the initial interaction of bacterial cellulose with the chitin component of the fungal cell wall.

## Results

### Biofilm formation and specificity

**Figure 1:**
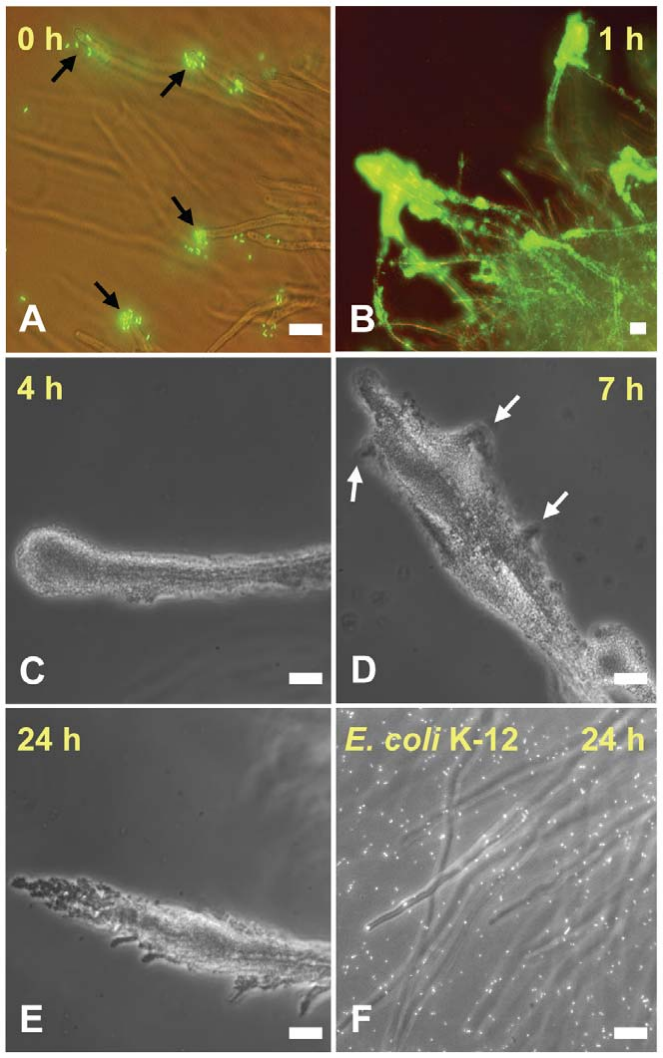
Time course of interaction of *S*. Typhimurium with *A. niger* hyphae. GFP-*S*. Typhimurium cells attached rapidly to the tip of the fungal hyphae (A, black arrows), where large biofilms formed within 1 h of incubation (B). The biofilm coated the entire hyphae by 4 h (C) and began to differentiate by 7 h (D, white arrows). Distinct branching of the biofilm was visible by 24 h (E). *E. coli* K-12 (stained with SYTO9®) did not display any significant attachment to the hyphae by 24 h (F). Scale bars, 20 µm.

Time-course microscopy revealed that *S.* Typhimurium strain SL1344 attached immediately to *A. niger* after the two organisms were mixed in KP buffer. The attachment started preferentially at the tip of the hyphae, but rapidly extended along the hyphae (Fig. 1A). Within one hour of incubation, *S.* Typhimurium aggregated in multiple cell layers on most protruding hyphae of the *A. niger* colony and formed a dense biofilm (Fig. 1B). Many planktonic cells were observed attempting to join the biofilm by very actively probing the outer biofilm layer. Large round aggregates were visible at the hyphal tips at four hours and bulges in the biofilm indicated the beginning of the branching of the biofilm (Fig. 1C). These further developed into distinct branches at seven hours of incubation (Fig. 1D) and by 24 h, the biofilms on the hyphae were fully branched with large aggregates hanging from the hyphal tips (Fig. 1E). The temporal biofilm development on this *A. niger* isolate from almond was similar to that observed with *A. niger* strain ATCC9029 (data not shown).

*S. enterica* serovars Enteritidis, Newport, and Thompson, all behaved similarly to serovar Typhimurium in mixed suspension with *A. niger* colonies. However, *E. coli* K-12 (Fig. 1F), *P. agglomerans,* and *P. chlororaphis* did not attach to *A. niger* hyphae. *E. coli* serovar O157:H7 EDL933 showed variable attachment on the hyphae, ranging from a few attached cells to none, and never produced a biofilm.

### Attachment to chitin and inhibition with N-acetylglucosamine (GlcNAc)

**Figure 2:**
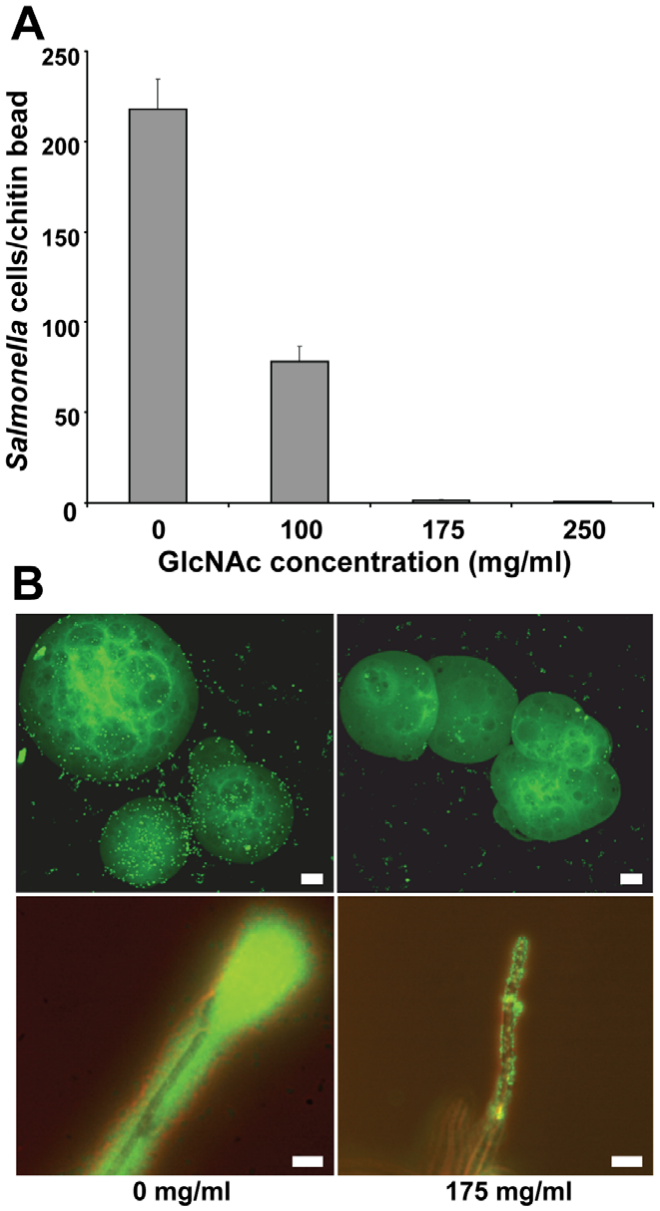
Inhibitory effect of pre-incubation of *S*. Typhimurium with GlcNAc on its attachment to chitin beads and *A. niger* hyphae. (A) Increasing concentrations of GlcNAc greatly inhibited the number of bacterial cells attached per chitin bead; mean number of cells per bead was 0.30 (s.e.m., 0.13) with 250 mg/ml GlcNAc. (B) Epifluorescence microscopy of bacterial cells pre-treated with 175 mg/ml GlcNAc revealed minimal binding to chitin beads (top right panel) and lack of biofilm formation on the hyphae (lower right panel), compared with the controls (left panels), as evidence by staining with SYTO 9®. Scale bars, 20 µm.

The fungal cell wall is composed of chitin, a polymer of β-1,4 GlcNAc [12]. The interaction between various bacterial species and *A. niger* observed above correlated well with their attachment to chitin beads. Namely, all four *S. enterica* serovars forming biofilms on *A. niger* also attached to the beads, whereas *E. coli* K-12, *P. agglomerans*, and *P. chlororaphis* failed to bind, and *E. coli* O157:H7 EDL933 displayed minimal attachment that did not exceed 10 cells per bead (Table 1). Incubation of *S.* Typhimurium cells with GlcNAc prior to exposure to chitin beads inhibited the attachment of the pathogen in a concentration-dependent manner. At the highest concentration of GlcNAc (250 mg/ml), the number of bacterial cells per bead decreased 727-fold compared with pre-incubation without GlcNAc (Fig. 2A and B, top panel). A similar inhibitory effect of GlcNAc was observed for attachment to *A. niger*, although binding was not fully blocked (Fig. 2B, lower panel). Other carbohydrates such as glucose, sucrose, and methylmannose did not have any effect on attachment to the fungus, while mannose increased binding considerably (data not shown).

**Table 1:** Attachment of various bacterial strains to chitin beads, including *S*. Typhimurium wild-type and derivative strains affected in cellulose synthesis.

Bacterial strain	Number of cells/bead^1^	Fluorescence on CFA^2^
*S. enterica* MB282 (WT)	245.00 (5.00)	++++
*S. enterica* MB664 (cellulose^-^ mutant)	0.20 (0.12)	-
*S. enterica* MB683 (complemented mutant)	194.20 (4.32)	+++
*E. coli* K-12	0.25 (0.12)	-
*E. coli* K-12 pMB682^3^	53.70 (3.26)	++
*E. coli* O157:H7	4.40 (0.76)	+
*P. agglomerans*	0.21 (0.10)	-
*P. chlororaphis*	0.20 (0.11)	-

1 Average calculated from 10 chitin beads in each of two replicate suspensions (s.e.m.).

2 Relative qualitative assessment of cellulose production assayed by fluorescence intensity of colonies on CFA-no salt due to binding of the bacterial cells to Calcofluor White.

3 Plasmid used for complementation of cellulose deficiency as in MB683; carries SL1344 cellulose synthesis operon.

### Role of cellulose

**Figure 3:**
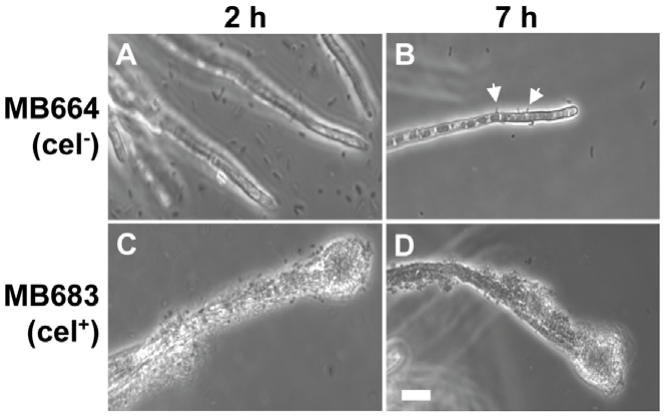
Comparative micrographs of the interaction of the cellulose-deficient *S*. Typhimurium strain MB664 (cel^-^, A and B) and its complemented strain MB683 (cel^+^, C and D), after 2 h (left panel) and 7 h (right panel) of incubation with *A. niger*. Strain MB664 failed to attach to the hyphae except for a few rare random cells at later time points (white arrows, B). Transformation with pMB682 restored attachment at high density and formation of a stable biofilm at later incubation times (C and D), albeit not as thick as that of the wild-type strain, as shown in figure 1D. Scale bar, 20 µm.

Because cellulose has been implicated in *Salmonella* biofilm formation [13], we examined the role of this exopolysaccharide in the ability of *S*. Typhimurium to bind to and aggregate on *A. niger*. Strain MB664, a mutant of SL1344 MB282 that is defective in cellulose synthesis, was unable to bind to chitin beads (Table 1) and only very rare individual cells attached to *A. niger* hyphae (Fig. 3A and B). This near complete lack of attachment to the hyphae resulted in the inability to aggregate and form a biofilm on the fungus. Transformation with pMB682, a plasmid encoding the cellulose synthesis operon from SL1344, was used to restore cellulose production and complement the mutation in strain MB664. The cellulose-complemented strain (MB683) regained attachment to chitin beads at 79% of the density of the wild-type strain (Table 1), and attached and aggregated on *A. niger*, albeit at slightly lower levels than the wild-type strain (Fig. 3C and D). *E. coli* K-12, which does not produce cellulose [13], is unable to bind to chitin beads and to *A. niger* (Fig. 1F and Table 1). Transformation with pMB682 conferred upon *E. coli* K-12 the ability to attach to chitin beads at 22% of the *S*. Typhimurium density (Table 1) and to form thin layered aggregates along the *A. niger* hyphae (data not shown).

**Figure 4:**
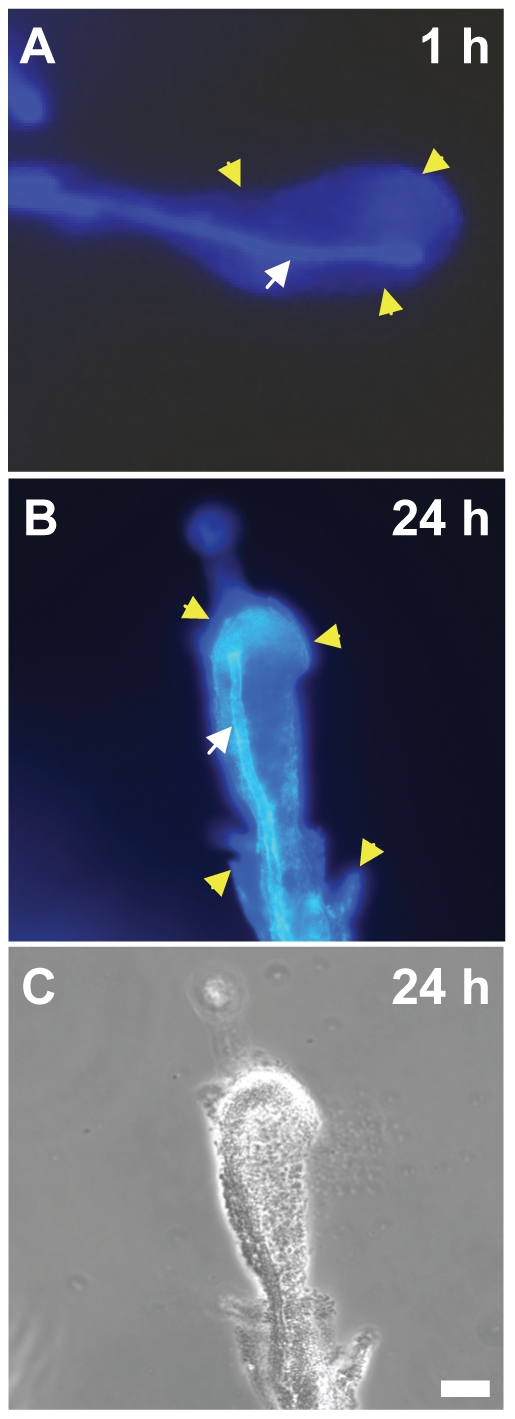
Detection of cellulose in biofilms on *A. niger*. Micrographs of *S*. Typhimurium biofilms on the fungal hyphae after 1 h (A) and 24 h (B and C) of co-incubation. Top and middle panel images show the fluorescence emitted from the chitin in the fungal cell wall (white arrows) and from the bacterial cellulose present in the biofilms (yellow arrows) after staining with Calcofluor White and UV illumination. Figure C is a phase contrast version of the epifluorescence image shown in B. Scale bar, 20 µm.

Calcofluor White binds to cellulose and therefore, colony fluorescence on Calcofluor White-containing LB-no salt agar (CFA-no salt) was used as a qualitative assessment of bacterial cellulose production, as previously described [13]. Whereas *S*. Typhimurium WT produced brightly fluorescent colonies on this medium, strains such as *E. coli* K-12, *P. agglomerans* and *P. chlororaphis,* which did not attach to chitin beads and to *A. niger* hyphae, produced non-fluorescent colonies similarly to the cellulose-deficient mutant of *S*. Typhimurium (strain MB664) (Table 1). Colonies of *E. coli* O157:H7 EDL933, which displayed little attachment to chitin beads and to *A. niger*, and was unable to form a biofilm on the fungus, showed very weak fluorescence on CFA-no salt (Table 1). Additionally, colony fluorescence was restored in the *S*. Typhimurium complemented cellulose-minus mutant, strain MB683. *S*. Typhimurium strain ATCC14028, which produced amounts of cellulose equivalent to those in SL1344 on CFA-no salt (Fig. S1), attached to *A. niger* to form mixed biofilms similarly to the latter strain (Fig. S2). Thus, the production of cellulose by the various bacterial strains tested for biofilm formation on *A. niger* in this study correlated well with their ability to attach to chitin beads and to the fungus. Additional evidence that cellulose is implicated in attachment and biofilm formation on the fungus was provided by the strong fluorescence resulting from staining with Calcofluor White of the aggregated *S.* Typhimurium cells one hour into their interaction with *A. niger* (Fig. 4A), and of the mature biofilm (Fig. 4B and C).

### Role of curli fimbriae

**Figure 5:**
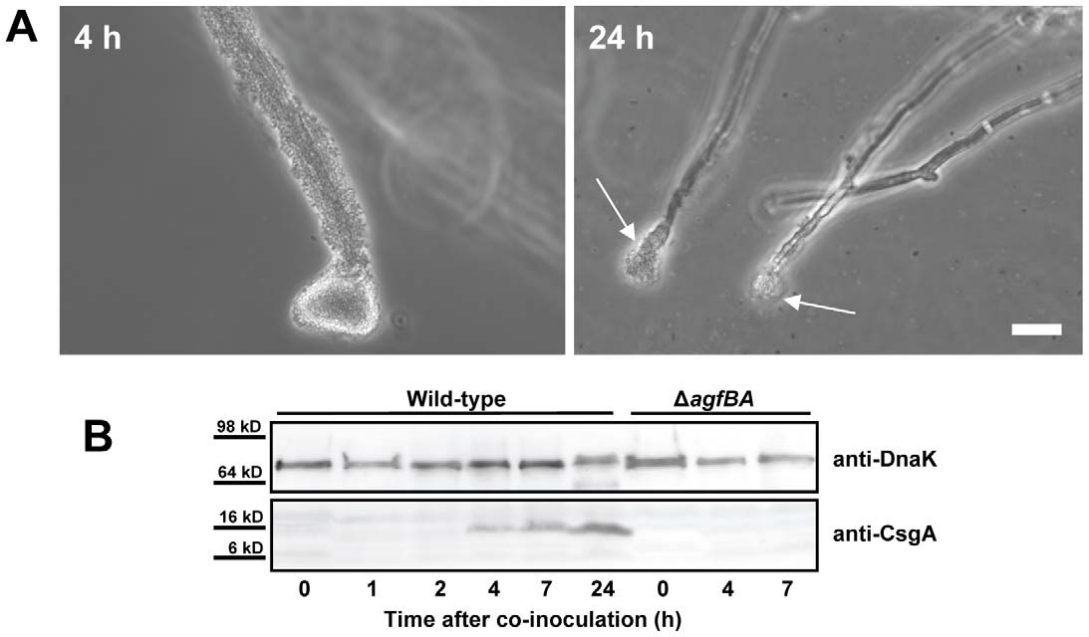
Role of curli in the interaction of *S*. Typhimurium with *A. niger*. (A) Colonization of the hyphae by a S. Typhimurium mutant deficient in curli fimbriae production (Δ*agfBA*). Note that the 4 h-biofilm formed by this mutant is thick and very similar to the WT biofilm shown in figure 1C. By 24 h, the mutant remains on the hyphae mostly at their tips (long white arrows) compared with the WT, which persists as a biofilm along the entire *A. niger* hyphal surface, as shown in figure 1E. (B) Whole-cell Western blot analysis of curli production by *S*. Typhimurium wild-type and its AgfBA-minus mutant during biofilm production on *A. niger*. The blot was probed with anti-DnaK antibody as an internal control (top panel) and with anti-*E. coli* CsgA antibody (lower panel), which also binds to AgfA. The time (h) of sampling during co-incubation is given under the lower panel. Samples at 0 h were obtained from *S*. Typhimurium inoculum cells grown in LB broth at 28°C. Curli were not detected in the whole-cell lysates of the Δ*agfBA* mutant at 24 h. Scale bar, 20 µm.

Curli are long aggregative fimbriae that mediate the attachment of *S*. *enterica* to various surfaces and have a role in biofilm formation [14]. Deletion mutants of *S*. Typhimurium in *agfD* and *agfBA*, which encode the transcriptional regulator of the curli operons and the curli structural proteins, respectively, were not altered in attachment during the early stages of the interaction with *A. niger* (Fig. 5A, left panel). However, the curli mutants were unable to persist in the biofilm on the hyphae as long as the wild-type cells since the curli mutant cells noticeably started to detach by seven h of incubation, leaving mostly barren hyphae with occasional cell aggregates at their tips by 24 h (Fig. 5A, right panel). As revealed by Western blot analysis using the antibody to *E. coli* CsgA (AgfA), the major curlin subunit AgfA was detectable in considerable amounts in the biofilm only after four h of incubation and its production increased up to at least 24 h (Fig. 5B). As expected, no antibody signal was detected from biofilm cells of the AgfBA-minus mutant strain, which retained the ability to colonize the hyphae up to seven h (Fig. 5B). A mutant deficient in MlrA (strain ℵ^8702^), an RpoS-dependent positive regulator of curli production [15], showed decreased persistence on the hyphae, similarly to the AgfBA-minus mutant (Fig. S3). Strain SL1344K [16], which is deficient in RpoS, a global regulator involved in curli gene expression [17] was more impaired in attachment and aggregation than the curli- and MlrA-minus mutants. This mutant attached to the sides of the hyphae only as small sparse aggregates while still forming large aggregates at the hyphal tips (Fig. S3), and failed to remain attached by 24 h of incubation, similarly to the curli-minus mutants (Figs. 5A and S3). It is noteworthy that *S.* Typhimurium strain LT2, which produces lower levels of RpoS than strains 14028 [18] and SL1344, behaved very similarly to SL1344K in its interaction with *A. niger* (data not shown). Since the abundance of RpoS is low under high nutrient conditions, it is also significant that *S*. Typhimurium-*A. niger* biofilms in rich culture medium, such as LB broth, were unstable similarly to those produced by the curli- and MlrA-minus mutants.

### Role of other fimbriae

*S*. Typhimurium ATCC14028 produces biofilms on *A. niger* similarly to strain SL1344. Therefore, available mutants in fimbrial operons *agf*, *bcf*, *fim*, *lpf*, *pef*, *stf*, *std*, *stb*, *sth*, and *stc* of strain ATCC14028 [19] were tested for attachment and biofilm formation on the fungus in comparison with the wild-type strain. None of the fimbrial mutants showed any detectable defect in attachment and biofilm formation on the hyphae within 4 h of incubation (data not shown), suggesting that these attachment factors do not have a primary role in the interaction of *S*. Typhimurium with *A. niger*.

## Discussion

Our study identifies an intimate interaction of *S. enterica* with *A. niger* that leads to complex biofilm formation and can dramatically impact the behavior of the pathogen in the environment and its epidemiology. Upon co-inoculation with *A. niger* mycelial colonies, *S*. Typhimurium rapidly colonized the hyphal tips and subsequently aggregated on the entire hyphal surface to form large multilayered and branched biofilms. The near complete lack of attachment of a cellulose-deficient mutant of *S*. Typhimurium to *A. niger* hyphae and to chitin beads, and recovery of binding to these surfaces in the complemented strain reveals that production of cellulose is essential for initial attachment to *A. niger* and to chitin, a structural component of fungal cell walls. Römling and co-workers have demonstrated that cellulose is a major constituent of the *S*. Typhimurium biofilm matrix [13]. Staining with Calcofluor White showed that cellulose was present in the *S*. Typhimurium biofilm throughout its development on the *A. niger* hyphae. Cellulose is synthesized by several bacterial species [20], [21], [22] and contributes to bacterial colonization of abiotic surfaces [13], eukaryotic cells [23], and plant tissue [24], [25], [26]. *E. coli* K-12, *P. agglomerans* and *P. chlororaphis* did not produce fluorescent colonies on CFA-no salt, and *E. coli* O157:H7 EDL933 colonies emitted only faint fluorescence, which is indicative of their low ability to synthesize cellulose, and correlates well with their poor binding to chitin beads and *A. niger* hyphae. Thus, differences in cellulose biosynthetic ability may account for variations in the interaction with *A. niger* among bacterial species and even between strains of a given species, such as *E. coli*.

Recent studies have reported a link between aspergillosis and the presence of opportunistic pathogenic bacteria in humans [27]. The possibility that other cellulose-producing bacterial pathogens may similarly interact with pathogenic *Aspergillus* species or other fungal genera and thereby aggravate human illness like cystic fibrosis or cutaneous infections, is of much relevance to medical microbiology. The critical implication of cellulose in such mixed biofilms would provide new potential avenues for disease treatment.

Chitin has been described as an important substratum for attachment and biofilm formation by *Vibrio cholerae* [28] and this association involves the GlcNAc-binding protein A (GbpA) [29], [30]. GlcNAc is the monomeric component of chitin and is therefore present in the fungal cell wall [12]. The competitive inhibition of attachment to chitin beads and hyphae by pre-incubation of *S*. Typhimurium cells with GlcNAc, and the loss of attachment to these surfaces in a *S*. Typhimurium cellulose-deficient mutant, suggest that chitin-cellulose interactions mediate the initial contact between the two organisms. Self-assembled polymers of cellulose and chitin have been produced in the laboratory [31] however, the chemistry of a putative cellulose-chitin interaction in our system remains unclear.

It is noteworthy that *S*. Typhimurium possesses several genes that potentially encode chitinases [32]. Chitinase activity has been reported in several human bacterial pathogens, and in some species, has been implicated in their virulence in mammalian hosts [33], [34], [35], [36], [37]. Although a change in the morphology or viability of the hyphae colonized by *S.* Typhimurium was not observed in our study, the possibility remains that chemical alterations of the fungal cell wall or membrane take place to the benefit of the pathogen cells in the biofilms. Further studies are needed to assess the role of chitinase homologs in the interaction of *S*. Typhimurium with *A. niger*.

Cellulose and the thin aggregative fimbriae, curli have a concerted role and linked regulation in biofilm production [14], [21], [38]. Our observations that the curli-deficient AgfD and AgfBA mutants of *S*. Typhimurium still attach to and aggregate on the hyphae at high densities, and that curli are produced late during biofilm formation indicate that these fimbriae are not essential for the initial stages of the interaction. Although curli fibers are required for primary adhesion to diverse surfaces [39], [40], their predominant role in later stages of biofilm formation has been reported in some systems [41], [42]. Likewise, the instability of the *S.* Typhimurium biofilms produced by mutants deficient in curli or in regulators affecting curli expression, such as RpoS and MlrA, suggests that these amyloids are important strengthening structures in biofilms formed by the pathogen in association with *A. niger*. Given that cellulose production in *S*. Typhimurium is post-transcriptionally controlled by AdrA, which is positively regulated by AgfD [13] and further upstream by MlrA [43], it would be expected that AgfD- and MlrA-minus mutants are deficient in attachment to *A. niger*, similarly to the cellulose-minus mutant. However, Garcia *et al*. demonstrated that whereas MlrA and AdrA control cellulose synthesis in complex culture medium, these are not required in nutrient-deficient medium; rather, GcpA acts as the post-transcriptional regulator for cellulose production under the latter conditions [43]. This differential regulation may explain the ability of the AgfD- and MlrA-minus mutants to attach to *A. niger* hyphae during the early stage of the interaction similarly to the wild-type strain, since cellulose production would be independent of these regulators under the low nutrient conditions of our system.

Our study reveals a critical role for cellulose in bacterial attachment to fungi, and to chitin in particular. It is likely that similar cellulose-mediated alliances of human pathogens with fungi or alternate chitinaceous hosts remain to be uncovered. Since chitin is an abundant polysaccharide in nature and is an outer-surface component in numerous organisms belonging to diverse phyla, the production of cellulose by pathogenic bacteria may represent an ecologically successful strategy for survival and dispersal via binding to abiotic and eukaryotic chitinaceous surfaces.

## Materials and Methods

### Strains and culture conditions

Bacterial and fungal strains used in this study are listed in Table 2. *S.* Typhimurium strain SL1344 was used for biofilm formation. An SL1344 cellulose-minus mutant (strain MB664) was generated using the λ Red recombinase system [44] by replacing *bcsB-bcsA-yhjQ* with a kanamycin resistance cassette. The complemented mutant strain MB683 was constructed by transforming strain MB664 with plasmid pMB682, which harbored the entire cellulose operon of SL1344 cloned into the SacI site of pBBR1MCS5 [45] in orientation of *lacZ* transcription. Primers used for mutagenesis and cloning are listed in Table S1. Cellulose synthesis was assessed by streaking bacterial strains onto LB-no salt agar containing 0.025% Calcofluor White (Sigma) (CFA-no salt) and testing for colony fluorescence under UV light after 48-h incubation at 28°C, based on the method by Zogaj *et al.* [13]. Strains MB430 and MB463 are curli-minus derivatives of SL1344 constructed by replacement of *agfBA* and *agfD*, respectively, with a kanamycin resistance cassette, using pSF31 [46] and pUMR5 [47] for allelic exchange, respectively. Absence of curli production was confirmed on Congo Red agar, as previously described [48]. All gene deletions and cloning were confirmed by PCR.

**Table 2:** Strains and plasmids used in this study.

Strains	Description	Source
*S*. Typhimurium SL1344		
MB282	Wild-type, strep^R^	Gift from S. Falkow
MB285	GFP-labeled MB282 via pGT-KAN; strep^R^ gent^R^	This study
MB430	MB282-derivative; Δ *agfBA*::Kan^R^ cassette; kan^R^	This study
MB463	MB282-derivative; Δ *agfD*::Kan^R^ cassette; kan^R^	This study
MB664	MB282-derivative; *bcsB-bcsA-yhjQ* replaced with a kan^R^ cassette; strep^R^ kan^R^	This study
MB683	MB664-derivative containing pMB682; strep^R^ kan^R^, gent^R^	This study
ℵ^8702^	Derivative of SL1344; *mlrA*:*:tetAR*	[15]
SL1344K	Derivative of SL1344; *rpoS::kan*	[16]
*S.* Typhimurium ATCC14028	Wild-type, smooth	Gift from E. Trees
*S*. Enteritidis LJH608	Isolated from outbreak-associated almonds	[11]
*S*. Newport 96E01153-C-TX	Isolated from outbreak-associated alfalfa seeds	[51]
*S*. Thompson 99A2345	Clinical isolate from outbreak linked to cilantro in California	[52]
*E. coli* K-12 ATCC29425	Wild-type K-12	ATCC
MB684	Wild-type K-12 ATCC29425 transformed with pMB682; gent^R^	This study
*E. coli* O157:H7 EDL933	Isolated from outbreak-associated hamburger meat	ATCC
*Pantoea agglomerans*		
MB42	Isolated from leaves of cilantro plants in California	[53]
*Pseudomonas chlororaphis*		
MB10	Isolated from leaves of cilantro plants in California	[53]
*Aspergillus niger* MB7	Isolated from almond kernels in California	Gift from S. Hua
**Plasmids**		
pGT-KAN	Broad-range vector with constitutively expressed *gfp*; gent^R^	[54]
pBBR1MCS-5	Broad-range cloning vector; gent^R^	[45]
pMB682	pBBR1MCS-5 carrying the cellulose operon of SL1344l; gent^R^	This study

#### Bacterial cultures

All strains were cultured in Luria Bertani (LB) broth at 28°C and 20 rpm until early stationary phase of growth. LB broth was amended as appropriate with streptomycin (30 µg/ml), kanamycin (50 µg/ml), gentamycin (15 µg/ml), or ampicillin (100 µg/ml). Inoculum cells were washed twice in potassium phosphate buffer (10 mM, pH 7.0) (KP buffer) and resuspended in the same. The final concentration of the bacteria in mixed suspensions with *A. niger* was 2×10^7^ cells/ml.

#### Fungal cultures

*A. niger* MB7 was isolated from almond kernels grown in California. Its identification was confirmed by partial sequencing of its 28S rDNA using *A. niger* primers described by Sandhu *et al*. [49]. *A. niger* was grown at 28°C on Potato Dextrose Agar with tetracycline (12 µg/ml) and cefoperazone (33 µg/ml) for two weeks. Spores were collected and their concentration quantified with a hemacytometer. Potato Dextrose Broth was inoculated at 10^3^ spores/ml and incubated overnight at 130 rpm and 28°C to allow for spore germination. One ml of the germinated spores was added to 400 ml M9 minimal medium containing 0.1% sucrose. The fungal culture was grown at 130 rpm and 28°C for 72 h, at which time small mycelial colonies that were 2–5 mm in diameter had formed, prior to co-incubation with *S*. Typhimurium.

#### Co-cultures

The colonies from the fungal culture were washed twice by collecting them on a Cellector™ sieve (100 mesh size) and resuspending them in KP buffer. The fungus was added at a final concentration of approximately 1 mycelial colony per ml to a 1-L suspension of *S.* Typhimurium at 2×10^7^ cfu/ml KP buffer. The co-culture was incubated at 28°C and 130 rpm for various periods of time prior to microscopic observation or protein extraction for Western blot analysis.

### Microscopy of *S*. Typhimurium - *A. niger* biofilms

The mixed bacterial and fungal suspension incubated in KP buffer as described above was sampled at 0, 1, 2, 4, 7 and 24 h after inoculation. The fungal colonies were spooned out with an inoculation loop onto microscope slides for observation of bacterial attachment and biofilm formation on the fungal hyphae. To test the effect of N-acetylglucosamine (GlcNAc) on their attachment to *A. niger*, *S.* Typhimurium cells were grown and washed as above, and resuspended at 2×10^7^ cells/ml in 0, 100, 175, and 250 mg GlcNAc per ml KP buffer. The suspensions were incubated for 15 min at 100 rpm, and at 12°C to prevent bacterial growth on GlcNAc, before they were added to *A. niger* colonies. Observations were made with a Leica DMRB microscope under phase contrast illumination, or epifluorescence with a GFP, fluorescein, or RGB filter set.

### Chitin Bead Assay

Chitin beads (New England Biolabs) were washed twice by centrifugation and resuspension in KP buffer. Cells from overnight bacterial cultures grown in LB broth at 28°C were washed three times in KP buffer. In a culture tube, 950 µl of 2×10^7^ bacterial cells/ml were mixed with 50 µl of beads and the suspension was shaken at 100 rpm and 28°C for 1 h. The beads were washed three times by allowing them to settle to the bottom of the tube, removing the upper liquid by pipetting, and adding KP buffer. The beads and bound bacteria were stained with SYTO 9® stain (Molecular Probes) and imaged under a TCS NT confocal scanning laser microscope (Leica Microsystems). The number of bacteria attached per bead was assessed for ten beads from each of two replicate suspensions under the epifluorescence microscope.

To test the effect of GlcNAc challenge on the attachment of *S.* Typhimurium to chitin beads, bacteria were treated with GlcNAc as described above. Then each suspension was mixed with chitin beads, incubated, stained, and the cell number per bead assessed, as described above.

### Western Blot Analysis

Western blot analysis was carried out as previously described [50] with a few modifications. The bacterial pellets were resuspended in 95% formic acid to depolymerize the curli fibers, and dried. The sample was resuspended in 150 µl 2xSDS loading buffer, sonicated, heated to 100°C, centrifuged, and proteins in the supernatant were separated by SDS-PAGE. The proteins were blotted onto a PVDF membrane, probed first with a rabbit polyclonal anti-*E. coli* CsgA antibody [50] and a mouse anti-DnaK antibody (LifeSpan BioSciences), which was used as an internal control for normalization of protein concentration across the samples. The blot was then incubated with secondary antibodies (alkaline phosphatase-conjugated goat anti-rabbit and rabbit anti-mouse (Thermoscientifc) for CsgA and DnaK, respectively. The blot was developed with the NBT/BCIP substrate kit (Sigma-Aldrich).

## Supporting Information

Figure S1 Cellulose production by *S*. Typhimurium strains as evidenced by fluorescence on CFA-no salt. The right panel depicts the fluorescence of each bacterial spot shown in the left panel. Strain SL1344 produced considerable quantities of cellulose (A and E), similarly to strain ATCC14028 (B and F), whereas the cellulose-deficient mutant of SL1344 (strain MB664) failed to produce detectable amounts (C and G). *E. coli* K-12, which lacks the ability to synthesize cellulose, was used as a control and did not fluoresce on CFA (D and H). The dotted circles in the fluorescent images G and H represent the approximate contour of the spots shown in the bright field images C and D, respectively. The bacterial spots were obtained by applying two µl of a suspension of 10^8^ cfu/ml of each strain onto CFA-no salt, followed by incubation at 28°C for 48 h. The spots were observed under a Leica MZ-FLIII fluorescence stereomicroscope (Leica Microsystems) and the images were captured with a Sony DKC5000 digital color camera (Sony Electronics).
(TIF)

Figure S2 Phase contrast micrograph of *S*. Typhimurium ATCC14028 biofilms formed on *A. niger* hyphae. Biofilms are shown after 1 h (A) and 2 h (B) of co-incubation in KP buffer at 28°C. Note that this *S*. Typhimurium strain which has the ability to synthesize cellulose, as shown in figure S1, produces biofilms on the fungus that are very similar to those of strain SL1344 shown in figure 1. Scale bars, 20 µm.
(TIF)

Figure S3 Colonization of *A. niger* hyphae by S. Typhimurium WT and mutants that are deficient in RpoS and MlrA, two regulators of curli expression. Note that the 4 h-biofilms formed by the WT (A) and the MlrA-minus mutant (B) were similarly very thick whereas the RpoS-minus mutant aggregated only as round balls at the hyphal tips (short white arrows) with a few patches along the hyphae (yellow arrows) (C). By 24 h, the WT persisted as a biofilm along the entire *A. niger* hyphal surface (D) whereas both mutants remained on the hyphae mostly at their tips (long white arrows) (E and F). Scale bar, 20 µm.
(TIF)

Table S1 DNA primers used in this study.
(DOC)
