# Supplementary figures and images for: Salmonella Biofilm Formation on Aspergillus niger Involves Cellulose – Chitin Interactions
Source: PLoS One. 2011 Oct 7;6(10):e25553. doi: 10.1371/journal.pone.0025553 (PMC3189214; doi:10.1371/journal.pone.0025553)

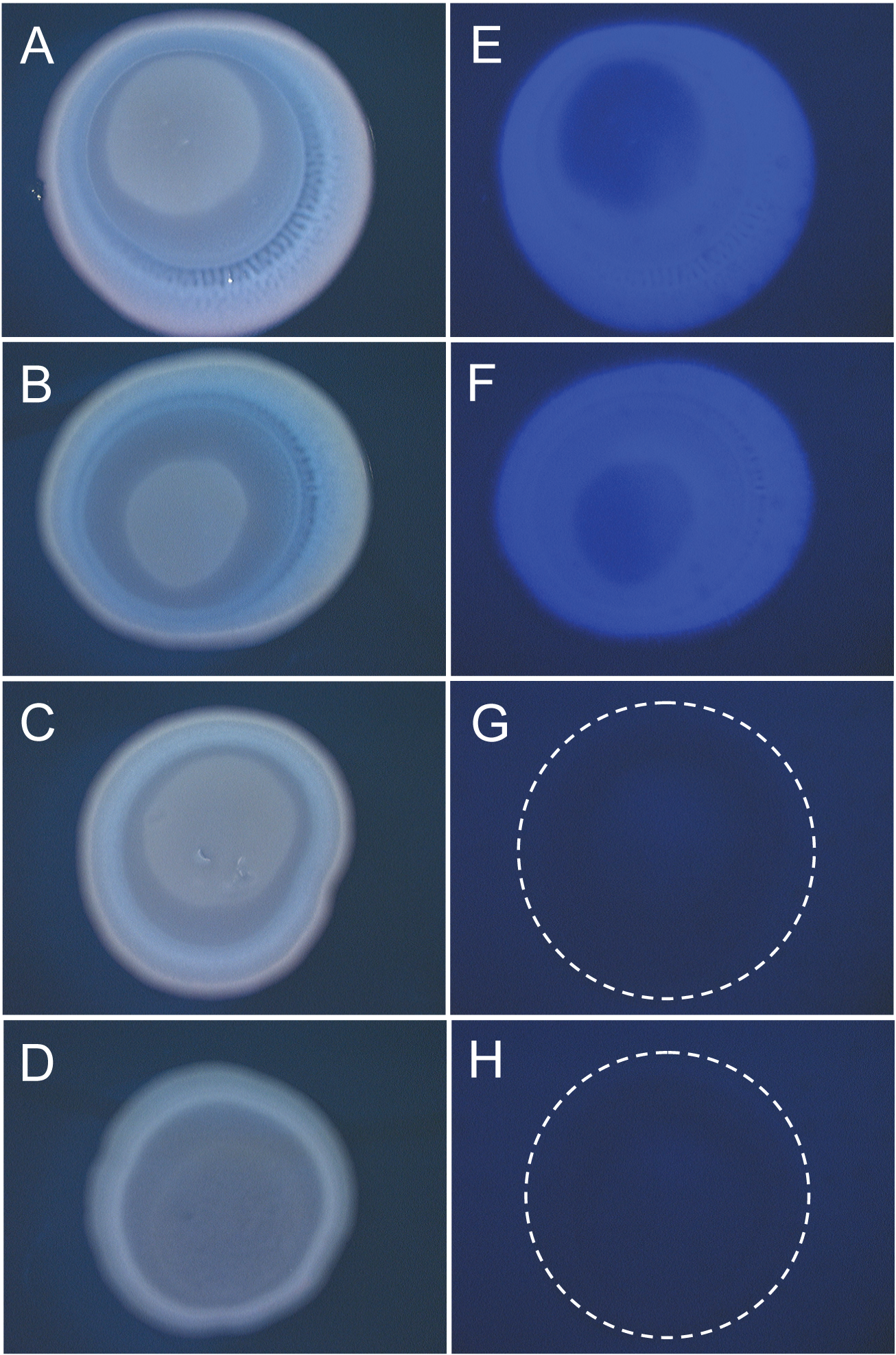

Supplement: Figure S1 — Cellulose production by S. Typhimurium strains as evidenced by fluorescence on CFA-no salt. The right panel depicts the fluorescence of each bacterial spot shown in the left panel. Strain SL1344 produced considerable quantities of cellulose (A and E), similarly to strain ATCC14028 (B and F), whereas the cellulose-deficient mutant of SL1344 (strain MB664) failed to produce detectable amounts (C and G). E. coli K-12, which lacks the ability to synthesize cellulose, was used as a control and did not fluoresce on CFA (D and H). The dotted circles in the fluorescent images G and H represent the approximate contour of the spots shown in the bright field images C and D, respectively. The bacterial spots were obtained by applying two µl of a suspension of 108 cfu/ml of each strain onto CFA-no salt, followed by incubation at 28°C for 48 h. The spots were observed under a Leica MZ-FLIII fluorescence stereomicroscope (Leica Microsystems) and the images were captured with a Sony DKC5000 digital color camera (Sony Electronics). (TIF) [file pone.0025553.s001.tif]

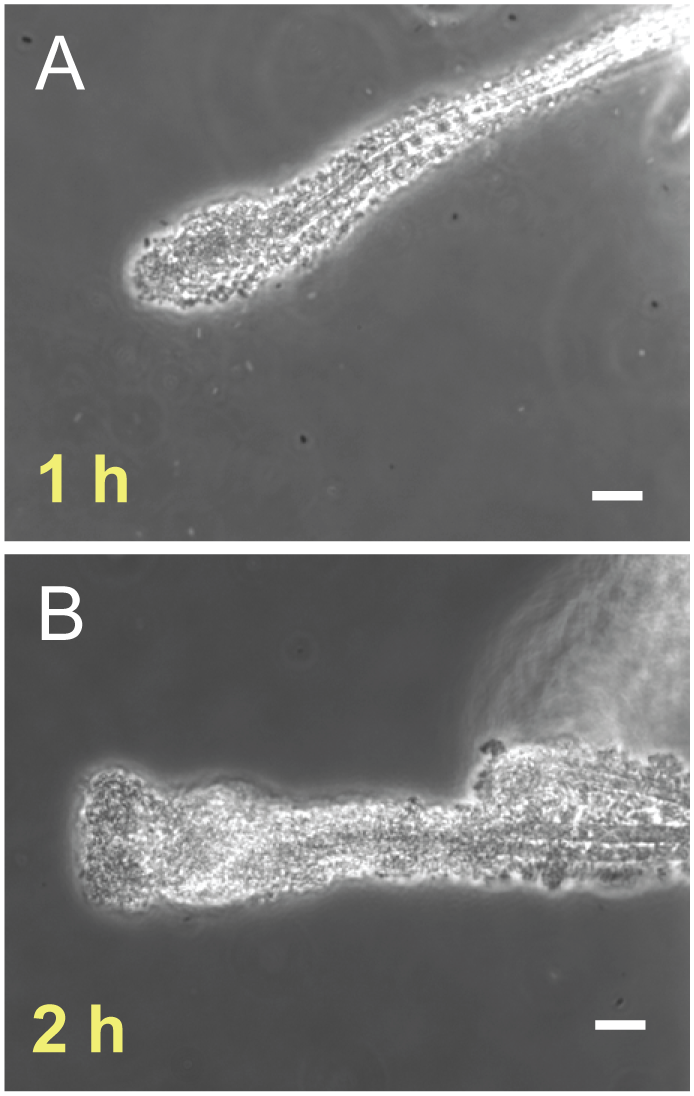

Supplement: Figure S2 — Phase contrast micrograph of S. Typhimurium ATCC14028 biofilms formed on A. niger hyphae. Biofilms are shown after 1 h (A) and 2 h (B) of co-incubation in KP buffer at 28°C. Note that this S. Typhimurium strain which has the ability to synthesize cellulose, as shown in figure S1, produces biofilms on the fungus that are very similar to those of strain SL1344 shown in figure 1. Scale bars, 20 µm. (TIF) [file pone.0025553.s002.tif]

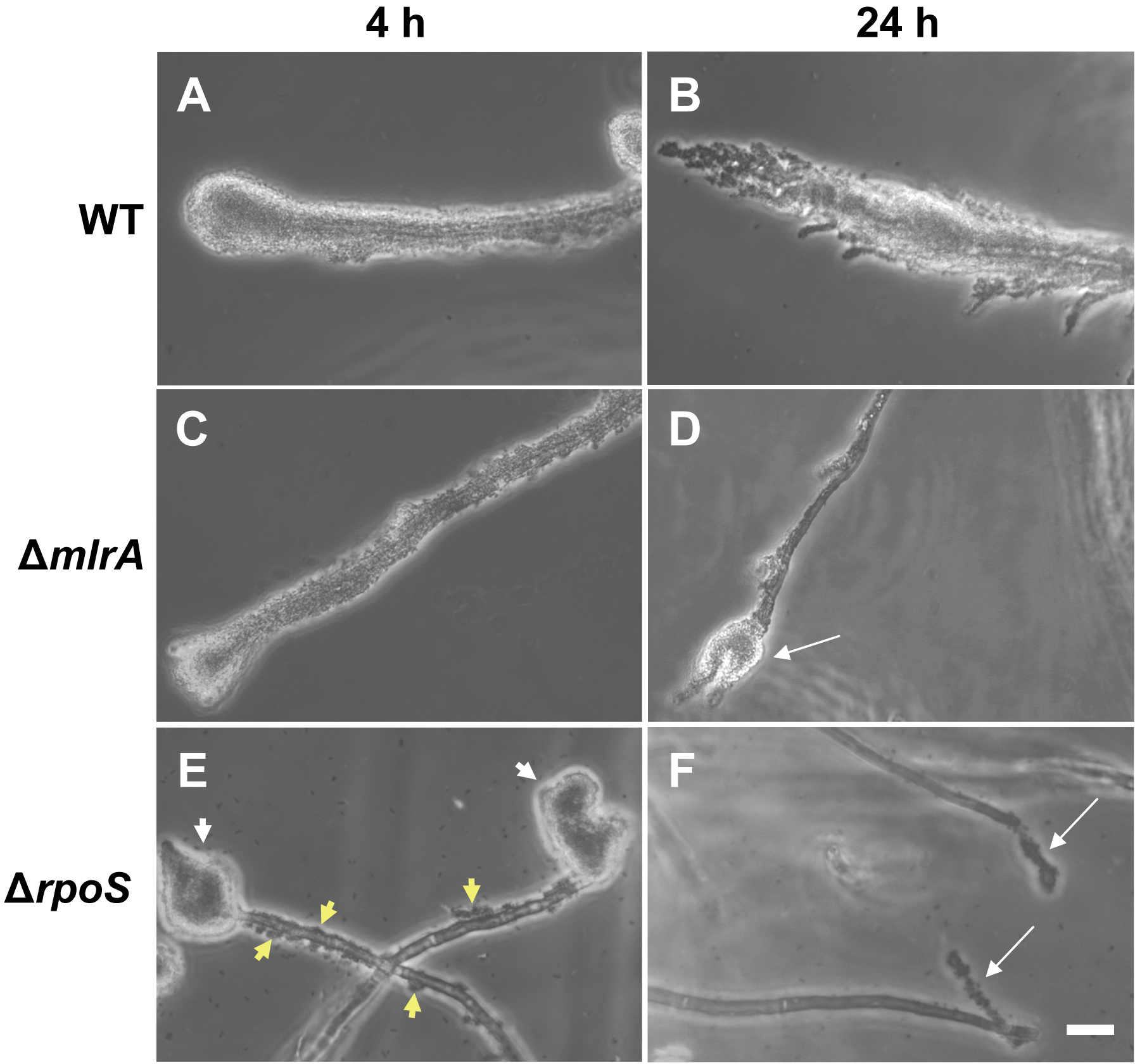

Supplement: Figure S3 — Colonization of A. niger hyphae by S. Typhimurium WT and mutants that are deficient in RpoS and MlrA, two regulators of curli expression. Note that the 4 h-biofilms formed by the WT (A) and the MlrA-minus mutant (B) were similarly very thick whereas the RpoS-minus mutant aggregated only as round balls at the hyphal tips (short white arrows) with a few patches along the hyphae (yellow arrows) (C). By 24 h, the WT persisted as a biofilm along the entire A. niger hyphal surface (D) whereas both mutants remained on the hyphae mostly at their tips (long white arrows) (E and F). Scale bar, 20 µm. (TIF) [file pone.0025553.s003.tif]
