# Supplementary material for: Salmonella Biofilm Formation on Aspergillus niger Involves Cellulose – Chitin Interactions
Source: PLoS One. 2011 Oct 7;6(10):e25553. doi: 10.1371/journal.pone.0025553 (PMC3189214; doi:10.1371/journal.pone.0025553)
Supplement: Table S1 — DNA primers used in this study. (DOC) [file pone.0025553.s004.doc]

**Table S1. DNA primers used in this study**

*Primers used for lambda Red-mediated cellulose-minus mutant construction*^a^:

| *yhjQ-*F  (H1+**P1**) | 5’TGCCTGCTGCATGATGCGGGCGACAAAACGTCCGCCGGGAGCCTGCGATG**GTGTAGGCTGGAGCTGCTTC**3’ |
| --- | --- |
| *bcsB-R*  (H2+**P2**) | 5’AGGACAATTTTCTTTTCATCGCATTATCATCATTGTTGAGCCTGAGCCAT**ATGGGAATTAGCCATGGTCC**3’ |

*Primers used for mutant complementation^b^*:

| Cellulose operon*-*F | 5’-*CCCC****GAGCTC***ACTAGCGAAAGCACAGAAATAAACCT-3’ |
| --- | --- |
| Cellulose operon*-*R | 5’-*CCCC****GAGCTC***AACCATTGCCATCTGTTTAATTGTTA-3’ |

^a^ Sequences complementary to pKD4 are in bold type, the rest of the sequence is homologous to the amplified gene.

^b^ Italicized sequences consist of an adaptor that includes the SacI restriction site in bold type.
